# Corrigendum

**DOI:** 10.1002/vms3.1103

**Published:** 2023-03-08

In the article by Talebi et al. (2023), the ethics code in the ethics statement section was incorrect. The correct code should read as “IR.LUMS.REC.1399.310”.

We apologise for the error.
